# Community interventions to prevent violence against women and girls in informal settlements in Mumbai: the SNEHA-TARA pragmatic cluster randomised controlled trial

**DOI:** 10.1186/s13063-019-3817-2

**Published:** 2019-12-17

**Authors:** Nayreen Daruwalla, Unnati Machchhar, Shanti Pantvaidya, Vanessa D’Souza, Lu Gram, Andrew Copas, David Osrin

**Affiliations:** 1 SNEHA (Society for Nutrition, Education and Health Action), 310, 3rd floor, Urban Health Centre, 60 Feet Road, Dharavi, Mumbai, Maharashtra 400017 India; 2 University College London Institute for Global Health, 30 Guilford Street, London, WC1N 1EH UK; 3 Institute of Clinical Trials and Methodology, 90 High Holborn, London, WC1V 6LJ UK

**Keywords:** Gender-based violence, Domestic violence, Intimate partner violence, India, Mumbai, Randomised controlled trial, Trial protocol

## Abstract

**Background:**

In a cluster randomised controlled trial in Mumbai slums, we will test the effects on the prevalence of violence against women and girls of community mobilisation through groups and individual volunteers. One in three women in India has survived physical or sexual violence, making it a major public health burden. Reviews recommend community mobilisation to address violence, but trial evidence is limited.

**Methods:**

Guided by a theory of change, we will compare 24 areas receiving support services, community group, and volunteer activities with 24 areas receiving support services only. These community mobilisation activities will be evaluated through a follow-up survey after 3 years. Primary outcomes will be prevalence in the preceding year of physical or sexual domestic violence, and prevalence of emotional or economic domestic violence, control, or neglect against women 15–49 years old. Secondary outcomes will describe disclosure of violence to support services, community tolerance of violence against women and girls, prevalence of non-partner sexual violence, and mental health and wellbeing. Intermediate theory-based outcomes will include bystander intervention, identification of and support for survivors of violence, changes described in programme participants, and changes in communities.

**Discussion:**

Systematic reviews of interventions to prevent violence against women and girls suggest that community mobilisation is a promising population-based intervention. Already implemented in other areas, our intervention has been developed over 16 years of programmatic experience and 2 years of formative research. Backed by public engagement and advocacy, our vision is of a replicable community-led intervention to address the public health burden of violence against women and girls.

**Trial registration:**

Controlled Trials Registry of India, CTRI/2018/02/012047. Registered on 21 February 2018. ISRCTN, ISRCTN84502355. Registered on 22 February 2018.

## Background

The Convention on the Elimination of All Forms of Discrimination Against Women was signed by 189 countries, including India, in 1980 [1]. The United Nations declared a response to violence against women and girls imperative in 2006 [2], and it was identified as a health priority in World Health Organization (WHO) guidelines of 2013 [3]. Elimination of violence against women and girls in public and private is a target for the fifth Sustainable Development Goal.

Globally, 30% (95% confidence interval [CI] 28, 32) of women have experienced physical or sexual violence by an intimate partner or sexual violence by a non-partner [4]. The question of how to achieve substantial reductions in violence against women in low- and middle-income countries is central to current debate [5]. The response needs to be multisectoral and to include both prevention and response, supported by research on the effects, costs, and potential scalability of promising interventions [6]. About 40% of survivors disclose violence—usually to a friend or relative—but only 7% to a formal source of support, making disclosure an early priority in a theory of change [7]. A recent systematic review of studies in India suggested that 29% of women had survived physical abuse in the past year, 22% had suffered psychological abuse, 7% sexual abuse, and 30% multiple forms of violence [8].

Violence causes non-fatal or fatal injuries: 21% of homicides in Southeast Asia are committed by an intimate partner, constituting 60% of all female homicides (the figure for male homicides is 1%) [9]. Other harms to health include sexually transmitted infections, miscarriage, induced abortion, stillbirth, low birth weight, preterm delivery, harmful drug and alcohol use, anxiety and depression, self-harm, suicide, and trans-generational recapitulation of violence [4, 10, 11]. Physical and psychological trauma and fear lead to mental health problems, limited sexual and reproductive control, somatoform conditions [4], difficulties in seeking healthcare, and lost economic productivity [12]. Violence is associated with male authority over female behaviour, justification of wife beating, and women’s economic disadvantage [13], all of which are manifest in India. Intimate partner violence is endemic, domestic violence extends beyond the WHO definition [14], to culturally sanctioned household maltreatment [15], and non-partner sexual violence is reported regularly in the media [16].

The latest National Family Health Survey (NFHS-4) suggests that 21% of ever-married women in Maharashtra state—the location of our work—have experienced intimate partner violence [17]. Risk factors for both physical and sexual violence include poverty, exposure to parental violence, childhood maltreatment, limited education, unemployment, young adulthood, mental disorder, substance use, individual acceptance of violence, weak community and legal sanctions, and gender and social norms supportive of violence [10]. These risk factors meet in Mumbai’s urban informal settlements, along with population density and stressful living conditions, and their toll in terms of violence is the reason for our activities. More than 377 million people live in urban India [18, 19]. Two-thirds of cities and towns include informal settlements [20], characterized by overcrowding, insubstantial housing, insufficient water and sanitation, lack of tenure, and hazardous location [21, 22]. There were an estimated ~ 105 million people in such settlements as of 2017 [19], and they currently include 41% of Mumbai’s households [20]. Women and girls in these communities lack both financial and social resources and also an understanding of the possibility of relief from endemic violence.

The SNEHA (Society for Nutrition, Education and Health Action) programme on Prevention of Violence against Women and Children began in 2000 and now includes ten counselling centres across Mumbai, linked with community mobilisation, health services, police, and legal support. The programme history follows global developments. The emphasis of a first wave of interventions, driven largely by feminist activism, was support for survivors of violence, reduction in secondary perpetration, strengthening legal recourse, and advocacy [23]. This led to the consolidation of services such as women’s shelters, counselling, legal advice, and laws such as India’s Protection of Women from Domestic Violence Act (PWDVA) 2005. A second wave of interventions, led by groups such as SNEHA, emphasises primary prevention and community activism and takes a public health position: population-based, interdisciplinary, and intersectoral [10].

The objectives of current efforts are both to respond to the burden of violence and to prevent it from happening [23, 24]. The underlying socio-ecological model locates individual personal histories within families, located in turn within communities, and in turn within societies [25]. There is broad agreement that interventions should operate at multiple levels, from individual to societal [26]. Interventional discourse has also moved from a concentration on the needs of survivors to an acknowledgment that intervention should aim to “transform the relations, norms, and systems that sustain gender inequality and violence” [27]. Of particular interest are interventions that aim to change norms that privilege controlling and aggressive masculine behaviour [27, 28]. Such interventions are usually termed ‘gender transformative’ [29], involve women, men, and young people [11], and aim to develop critical mass among community members, leaders, and institutions to change discriminatory social norms, promote gender equality, and reduce violence [24].

To achieve these aims, our preventive activities—the complex intervention that the trial will test—involve two kinds of community outreach: group education and enablement, and individual voluntarism. Group education involves women, men, and adolescents. It aims to develop awareness and understanding of violence, knowledge of rights and recourse, individual and collective local strategies for primary and secondary prevention, and increased confidence and leadership, and to reduce community tolerance and increase bystander action. Individual intervention involves women volunteers, *sanginis*, who identify survivors of violence, provide support, connect them with crisis intervention and counselling services, and facilitate police and health service consultation. *Sangini* response is supported by an innovative mobile electronic platform, Little Sister, which integrates real-time field reports of violence and their interventions with programme services. Our processes increase the social standing and agency of group members and *sanginis,* digital literacy, employability, and supportive social networks.

Secondary interventions for survivors—the background activities that will be available to both intervention and control groups in the trial—include counselling, liaison with the police, medical attention, mental health intervention, family interventions, and legal recourse. Our centres offer support from trained counsellors, clinical psychologists, municipal clinicians, visiting psychiatrists, and lawyers. SNEHA is a service provider under the PWDVA and runs women’s outpatient departments in three tertiary and one peripheral municipal hospital. We work with the police in five zones, training cadets and officers, and co-developing, piloting, and introducing guidelines for response to violence against women and girls into police practice. Components of our model have been adapted and replicated in collaborations with Ekjut in Jharkhand state and with Swasti in five states.

## Objectives

We aim to help people understand the gendered nature of violence, so that survivors make decisions, potential perpetrators think again, and others believe that action is possible. As a result of this, people will stand up against violence, individually and collectively, and community members will act to help survivors, will stop accepting violence, and will strengthen community structures that support a conviction that it is unacceptable. Our hypotheses are that women and girls will be more likely to disclose violence, that communities will become less tolerant of it, and that the prevalences of intimate partner and domestic violence will diminish.

### Primary hypothesis

Over and above a package of crisis intervention, counselling, and support services, a community mobilisation intervention delivered in informal settlements for 3 years and involving groups and volunteers will reduce the reported prevalence of domestic physical or sexual violence, and of domestic emotional or economic violence, control, or neglect.

### Secondary hypothesis

Over and above a package of crisis intervention, counselling, and support services, a community mobilisation intervention delivered in informal settlements for 3 years and involving groups and volunteers will increase the disclosure of intimate partner or domestic violence to support services, improve indices of community attitudes towards violence against women and girls, reduce the prevalence of non-partner sexual violence, and improve women’s mental health and subjective wellbeing.

## Methods

### Trial design

We will test the effects of community mobilisation through groups and volunteers in a parallel-group, phased, cluster randomised controlled pragmatic superiority trial, with 1:1 allocation to intervention and control in a total 48 urban informal settlement clusters.

### Setting

**Fig. 1:**
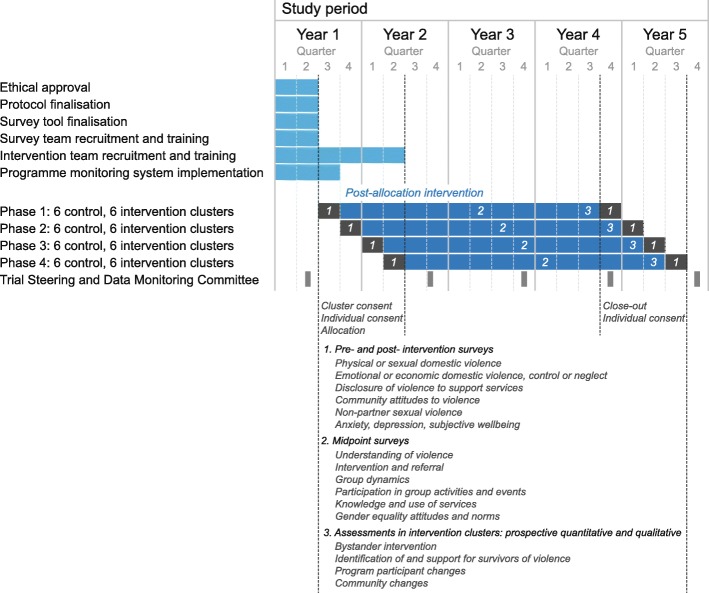
Trial timeline (adapted SPIRIT diagram)

We will select 48 informal settlement (slum) clusters, each of 500 dwellings, in Mumbai after vulnerability assessment. We will allocate 24 clusters randomly to receive the intervention and 24 to control. The trial will be implemented in four phases, each including six intervention and six control clusters. Each phase will begin with a pre-intervention survey. The intervention will be implemented for 3 years in each phase, followed by a post-intervention survey (see Fig. 1).

### Eligibility for intervention

Any resident of an intervention cluster may participate in the intervention. Women, men, and adolescents will be eligible to participate in group activities, and women will be eligible to volunteer as *sanginis*.

### Eligibility for evaluation

Two surveys will be administered before the intervention, two at the midpoint of the intervention, and two after the intervention. Two surveys will be used because one will examine women’s experience of violence and the other will examine attitudes about gender and violence in both women and men. An individual participant will respond to only one survey. We piloted both pre-intervention surveys with ~ 400 participants in areas similar to the trial clusters. Data collectors met with no significant problems. We will do a similar thing for the surveys at midpoint and after the intervention.

*Baseline survey 1* will ask 100 women aged 18–49 years in each of 48 clusters of 500 households about their health, wellbeing, common mental disorders, household decision-making, household power and control, neglect, experience of economic, emotional, physical, and sexual violence, disclosure and support (4800 participants).

Inclusion criteria are women aged 18–49 years who consent to interview.

*Baseline survey 2* will ask 50 women and men aged 18–65 in each of 48 clusters about gender roles, gender equality, ambivalent sexism, the problem of violence in their home area, attitudes towards and justifiability of violence against women, bystander intervention, and potential sources of support (2400 participants).

Inclusion criteria are women or men aged 18–65 years who consent to interview.

Midpoint surveys 1 and 2 will be administered after 1.5 years of intervention.

*Midpoint survey 1* will ask 20 women aged 18–49 years in each of 24 intervention clusters who are members of a women’s group about their understanding of violence, referral and intervention, group dynamics, and participation in group activities (480 participants).

Inclusion criteria are women aged 18–49 years who are members of women’s groups and consent to interview.

*Midpoint survey 2* will ask 20 women aged 18–49 years in each of 48 clusters about their knowledge of, attitudes towards, and participation in groups and events, knowledge of and use of services, as well as attitudes and norms around gender equality and violence (960 participants).

Inclusion criteria are women aged 18–49 years who consent to interview.

Post-intervention surveys 1 and 2 will be administered after 3 years of intervention. *Post-intervention survey 1* will be administered to 150 women in each cluster (7200 participants). The inclusion criteria will be the same as for pre-intervention (baseline) survey 1, with the addition of questions on exposure to the SNEHA-Taking Action, Reaching All (TARA) intervention. Post-intervention survey 2 will be administered to 50 women and men in each cluster (2400 participants). The inclusion criteria will be the same as for pre-intervention (baseline) survey 2.

### Other data collection within the trial

Theory-driven evaluation will include quantitative data from the baseline and post-intervention surveys, quantitative monitoring data, and qualitative data. These qualitative data will be collected by social scientists and ethnographers on the project team. They will involve interviews with, and observation of, programme participants and team members. Monitoring information will be collected by programme team members from salaried employees and volunteer men and women involved in the intervention. Included will be information on start-up processes, group attendance and meeting content, facilitator performance, community campaigns, and individual and group actions in the community. Exercises with group members will include time expenditure assessments to inform an economic evaluation (*separate protocol*) and questionnaires about collective efficacy arising from group work.

### Control arm activities

Like residents of areas outside our current programme, residents of control clusters will have access to our institutional services for crisis intervention and counselling. Control clusters will receive all SNEHA services, from existing centres in Mumbai, apart from community mobilisation activities: counselling, police liaison, medical attention, mental health intervention, family interventions, and legal recourse. Data collectors’ duty of care will extend to control clusters, from which women and girls will be referred for support with no restrictions if they disclose concerns about violence during data collection.

The SNEHA programme on Prevention of Violence against Women and Children began in 2000. It now includes five counselling centres and four centres at secondary and tertiary hospitals across Mumbai, all with access to in-house lawyers. It works with the police in five jurisdictions, has co-developed guidelines, and trains police cadets and officers to respond to violence against women and children. Having satisfied criteria for counselling, shelter, legal aid, and access to medical care, SNEHA is a service provider under India’s PWDVA 2005 and is able to file Domestic Incident Reports with legal validity. Under the Protection of Children from Sexual Offences (POCSO 2012), SNEHA has reporting rights to the police and Child Welfare Committee.

### Intervention arm activities

The trial will test a combination of group and individual community mobilisation activities on the background of secondary support services and infrastructure. Interventions will be implemented by salaried community organisers (women with higher secondary education, based at community centres), programme officers (women or men with graduate education), trained women counsellors, and programme coordinators (women or men with postgraduate education). In each cluster, we will convene three women’s groups, one men’s group, and one mixed-sex adolescent group, their members recruited through a community microplanning exercise employing participatory learning and action techniques. Groups will have 12–15 members and will meet twice monthly (women’s groups) or monthly (men’s and adolescent groups) for 1–2 h. They will follow manuals for sequential 1-year series that will iterate as participants develop over the 3-year programme. Year 1 will emphasise awareness and knowledge, Year 2 local action, and Year 3 leadership.

Women’s groups will be facilitated by community organisers. Meeting content has been developed from our experience and material from Raising Voices (SASA!), Stepping Stones, and Medica Mondiale. The sequence covers vision building, communication, understanding sex and gender, social norms, types of gender-based violence, response to violence, and legal support. Men’s groups will be facilitated by male programme officers and coordinators. Meeting content has been developed from our experience and material adapted from Yaari Dosti, Promundo, and Samyak. The sequence covers gender, sexuality, gender-based violence, leadership, legal awareness, and connections with police. Adolescent groups will be facilitated by programme officers and coordinators. Meeting content has been developed from our experience and material from Advocates for Youth, the Eberly Center for Teaching Excellence, the Ministry of Human Resource Development Adolescence Education Programme, and Planned Parenthood. The sequence covers self-awareness, sexual health and hygiene, gender and sexuality, gender-based violence, negotiating relationships, community participation, and mental health. Campaigns are prominent in our adolescent activities, including drama and dance. Neighbourhood events will be held twice a year and will include activities such as street theatre, games, film screenings, and mini-lectures. For each of the four phase areas, we will convene an offsite workshop twice a year for members of all groups to attend. Workshops will emphasise movement building against violence, technical knowledge on issues such as finance and government schemes, and interaction with the police and health services.

Individual activities will focus on *sanginis*: women who show leadership identified during women’s groups and campaigns, former clients, or women who have supported them. *Sanginis* will meet monthly (an awareness session and a general meeting) under the supervision of a programme officer. Awareness session content has been developed from our experience and material from Point of View and Medica Mondiale. The sequence covers personal strengths and weaknesses, sexual violence, mental health, counselling, safety measures, and connections with police. General sessions are structured around case discussions, requirements for organisational help, and identification of further training needs. *Sanginis* will be trained in the use of the *Little Sister* smartphone-based alert and follow-up system (https://www.ndtv.com/video/news/every-life-counts/with-support-from-an-app-women-stand-up-to-domestic-violence-in-dharavi-418956). They will undertake identification, crisis intervention and preliminary counselling, support, referral, and collective community campaigns. Community organisers and project officers will record their activities to contribute to programme monitoring and process evaluation, using electronic tablets and netbooks. The intervention will be described according to the Template for Intervention Description and Replication (TIDieR) checklist [30].

### Primary outcomes, comparing intervention and control arms, measured in cross-sectional survey after 3 years of intervention

The primary outcomes are:

1. Prevalence of physical or sexual domestic violence against women 15–49 years in the preceding 12 months, based on WHO, Indian National Family Health Survey (NFHS), and International Violence Against Women Survey perpetration modules. These are WHO consensus priority indicators (www.who.int/reproductivehealth/topics/violence/vaw-indicators/en/).
2. Prevalence of emotional or economic domestic violence or gender-based household maltreatment of women 15–49 years in the preceding 12 months, based on Indian NFHS and WHO modules and the new Indian Family Violence and Control Scale [31].

### Secondary outcomes, comparing intervention and control arms, measured in cross-sectional survey after 3 years of intervention

The secondary outcomes are listed as follows:

1. Proportion of violence against women and girls disclosed to support services (non-governmental organisations [NGOs], police, healthcare, government programmes)
2. Community tolerance of violence against women and girls:
  - Attitudes towards domestic violence
  - Attitudes towards gender equality
  - Attitudes towards rape and sexual violence
  - Bystander attitudes
3. Prevalence of non-partner sexual violence in preceding 12 months, based on Demographic and Health Surveys (DHS) and WHO modules
4. Prevalence of anxiety (Generalized Anxiety Disorder, 7 item questionnaire [GAD-7]) [32] and depression (Patient Health Questionnaire, 9-item [PHQ-9]) [33]
5. Subjective wellbeing (Short Warwick-Edinburgh Mental Wellbeing Scale [SWEMWBS]) [34]
6. Either or both of primary outcomes 1 and 2.

### Intermediate theory-based outcomes, measured in intervention clusters through prospective quantitative and qualitative data collection

The following intermediate theory-based outcomes are also measured:

1. Bystander intervention
2. Identification of and support for survivors of violence:
  - Survivors are identified, counselled, and referred.
  - Survivors access medical, police, and legal services.
  - Survivors understand violence against women and take action to prevent or respond to it.
  - Perpetrators understand abuse and others’ concerns.
  - Abuse is not private, others know about it, and families and friends seek help for violence.
  - Survivors feel less alone, more empowered, and have better mental health.
  - Survivors change their domestic situations.
3. Programme participants change:
  - People join groups or form more groups and networks with collective agency.
  - People discuss gender roles and violence and develop confidence to challenge norms.
  - People become leaders or change agents and bear witness to violence.
  - People trust our organisation, police, legal, and medical services.
4. Communities change:
  - Visible response to events leads to enquiries and awareness of programme activities.
  - Communities identify and report violence against women and referrals for early intervention increase.
  - Communities support women and impose sanctions against violence.

### Participant timeline

The participant timeline is summarised in Fig. 1. The four phases of the trial will include pre-intervention surveys, 3 years of intervention, and post-intervention surveys.

### Sample size

A cross-sectional sample of 100 women in each of 48 clusters at baseline and 150 at follow-up provides more than 80% power at 5% significance level to detect a minimum difference of 6% between arms in 12-month prevalence of domestic violence, reflected in either primary outcome. This minimum difference is considered conservative because of the 3-year intervention duration. Our power estimates are based on a range of intracluster correlation coefficients (ICCs) around 0.02 assumed to apply at both baseline and follow-up, for values of cluster autocorrelation ranging from conservative (0.5) to realistic (0.8), and a range of prevalence values for the control arm at follow-up around 15% for physical or sexual violence in the preceding 12 months and 80% for emotional or economic domestic violence, control, or neglect. Values assumed for the ICC and prevalence of each outcome are derived from a preliminary analysis of baseline data, pooling data from both arms. In an analysis of 5122 records (~ 100 per cluster), mean and median cluster prevalence of physical or sexual domestic violence in the preceding 12 months was 14% (minimum 2%, maximum 29%, interquartile range 10–17%). The ICC was 0.02 (95% CI 0.01, 0.03).

### Recruitment

Forty-eight clusters of 500 households each will be mapped and potential participants identified by visiting each house. Sampling will be by systematic interval with random start. Each participant will be visited by a field data collector to discuss the process, explain the trial, and receive a participant information sheet.

### Allocation sequence generation

Allocation will be through computer-generated pseudorandomisation, in four blocks corresponding to the four phases of implementation.

### Allocation concealment mechanism

Allocation will be done using cluster numbers that mask the allocator to geographical cluster identity.

### Implementation of allocation

Allocation will be done by a senior researcher (absent the trial statistician). The numerical allocation list will be matched with cluster name and location lists by the evaluation manager.

### Masking

Trial analysts will be masked to the association between numerical allocation of clusters and the names and locations of the clusters themselves. They will also be masked to allocation arm for interim and final analyses.

### Data collection

The TARA trial addresses questions in three broad categories, based on a published theory of change [35]:

1. *Effects*. Did the intervention work? Were effects seen on the primary, secondary, and intermediate outcomes specified in the theory of change?
2. *Resources and activities*. How well was the intervention done? What were the fidelity, reach, and quality of the activities?
3. *Explanation*. How did the activities achieve—or fail to achieve—the changes specified in the theory of change?

The trial will use data from eight sources:

1. A cross-sectional quantitative survey with women after 3 years of community intervention delivery, including questions about physical and mental health, experience of violence, disclosure to others, and help-seeking
2. A cross-sectional quantitative survey with women and men after 3 years of community intervention delivery, including questions about gender issues, attitudes towards violence against women and girls, bystander intervention, collective efficacy, and social capital
3. A cross-sectional quantitative survey with 15 women group members after 1.5 years of community intervention delivery, including questions about their understanding of violence, referral and intervention, group dynamics, and participation in group activities
4. A cross-sectional quantitative survey after 1.5 years of community intervention delivery with 20 women per cluster about knowledge of, attitudes towards, and participation in groups and events, knowledge and use of services, as well as attitudes and norms around gender equality
5. A management information system (MIS) recording group meetings, individual and collective community actions, casework, referral, counselling and psychologist support, and campaigns and community events
6. Qualitative research in intervention clusters, i.e. a series of purposively sampled case studies using ethnographic and qualitative methods such as participant observation, in-depth interviews, and focus group discussions with participants and groups, designed to contribute to the formulation of theory about intervention context, mechanisms, and outcomes [36–38]
7. A battery of assessments of the quality of mobilisation, facilitation, group processes, and supervision, collected annually for 3 years
8. A cohort of survivors of violence, with or without associated mental health concerns, recruited at the point of service consultation and followed up with individual interviews.

### Data management

Data will be entered at source into an electronic database, using Android tablets running Dimagi CommCare (https://www.dimagi.com/commcare/). The front end will incorporate display conditions and field constraints to optimise quality. Field supervisors will observe 5% of interviews, 10% of uploaded forms will be checked, and online databases will be downloaded daily for back-up. Data will be collected and stored by SNEHA, under an agreement with University College London (UCL), and in accordance with the UK Data Protection Act 1998 and the General Data Protection Regulation 2018.

### Statistical methods for primary and secondary outcomes

We will present a trial profile and comparisons of allocation group characteristics and trial indicators at baseline and follow-up. Proportions of primary and secondary outcomes will be reported by allocation group. Primary intention-to-treat analysis of intervention effect will be based on baseline and post-intervention data combined, using logistic regression with random effects for clusters. No formal adjustment to the standard 5% significance level will be made on the basis of two primary outcomes. We expect a residential turnover of ~ 25% per annum, addressed in ancillary ‘per protocol’ analyses of subgroups resident over the trial period. An analysis plan will be developed, examined by the Trial Steering Committee (TSC), and sealed prior to completion of data collection.

### Additional analyses

#### Effects: did the intervention work? Were effects seen on the primary, secondary, and intermediate outcomes specified in the theory of change?

Based on the programme theory of change, Additional file 1: Table S1 summarises the indicators we will use to evaluate effects and how we will collect them.

#### Resources and activities: how well was the intervention done? What were the fidelity, reach, and quality of the activities?

Based on the programme theory of change, Additional file 2: Table S2 summarises the indicators we will use to evaluate delivery, fidelity, quality, and the range and degree of responses to the community mobilisation intervention, and how we will collect them.

#### Explanation: how did the activities achieve—or fail to achieve—the changes specified in the theory of change?

##### Year 1: understanding context

A context document will describe the circumstances in which and people with whom intervention activities take place. Sources of information are summarised in Additional file 3: Table S3.

##### Year 2: developing hypotheses about what worked, for whom, and in what circumstances

Taking account of the contextual constructs identified in Year 1 (Additional file 2: Table S2), we will take a realist approach to developing hypothetical context-mechanism-outcome (CMO) configurations [39]. The means by which we test these configurations will be method-neutral, combining quantitative intervention monitoring data with qualitative data from interviews and observation of community activities.

##### Year 3: testing and refining hypotheses

We will test the candidate CMO configurations developed in Year 2 using a combination of quantitative and qualitative data. Beginning in Year 1, we will have developed longitudinal case studies in groups and neighbourhoods, and we will combine these with quantitative information on group attendance, activities, and outcomes.

#### Data monitoring

The trial will be governed by a combined independent TSC and a Data Monitoring and Ethics Committee (DMEC), and a SNEHA-UCL Trial Management Group (TMG), following Medical Research Council (MRC) and DAta Monitoring Committees: Lessons, Ethics, Statistics (DAMOCLES) terms of reference [40, 41]. The TSC will meet annually to provide oversight [42, 43]. The first meeting will take place after ethical approval and before recruitment begins, to review the protocol and establish the DMEC. Subsequent meetings will be annual. Information provision and TSC reporting templates will be used [43]. The DMEC will also meet annually [40]. We will undertake interim analyses for the DMEC. No stopping rule is envisaged. Charters for committees can be provided on request to the principal investigators.

#### Harms

The backbone of the trial is the provision of an extensive range of support services for survivors of violence. All investigators and intervention team members will be trained to respond to adverse events according to existing organisational protocols. Community organisers, supervisors, and interviewers will follow these protocols for response to survivors of domestic violence, non-partner sexual violence, or child sexual abuse. The protocols include immediate response (active listening, safety assessment, assessment of suicidal ideation, validation and support, psychological first aid, consideration of shelter, admission to hospital if necessary), enlisting of immediate help from line managers, doctors, or the police if required, and then provision of information and referral to an in-house counsellor. Subsequent arrangements encompass legal support, psychologist assessment and mental health counselling, and follow-up. The response to intervention activities may also follow ‘dark logic’ [44], elaborated in the theory of change. A woman’s disclosure of violence may lead to reprisals and a short-term increase in abuse, our activities may trigger vigilantism and precipitate action, people may demand that we support the perpetrator rather than the survivor, the wrong person may be punished, perpetrators may change the type of abuse they are enacting, or (ostensibly protective) limits may be set to women’s mobility. There is a small but important possibility of threats to team members and family or community hostility. We have substantial experience in dealing with each of these eventualities, including protocols for response to threat, in-house lawyers, and strong connections with the police.

#### Ancillary and post-trial care

Any participant or non-participant in trial areas will have free and open access to the full range of SNEHA support services.

## Discussion

A recent set of systematic reviews of interventions to prevent violence against women and girls developed a typology of effectiveness and the quality of evidence for it. Community mobilisation was rated as a promising population-based intervention [45], and included participatory projects and community-driven development engaging multiple stakeholders and addressing gender norms. Group-based training or workshops for prevention of violence against women and girls included empowerment training (rated as promising), work with men and boys on norms (rated as conflicting), and community workshops to promote changes in norms and behaviour (rated as promising) [23].

We lack adequately powered trials of clearly defined interventions of sufficient duration to have meaningful effects. We have identified 13 randomised trials of interventions to reduce domestic violence against women and girls in low- and middle-income countries. Six had urban components [46–51], 11 were in Africa [46–50, 52–57], and one in South Asia [51]. Seven of the trials combined violence prevention activities with HIV prevention [46–48, 50, 52, 56, 58], and four with enterprise and microfinance [52, 53, 55, 58]. In South Africa, the Intervention with Microfinance for AIDS and Gender Equity (IMAGE) study added to microfinance Sisters for Life, a 10-session programme followed by community mobilisation to engage adolescents and adult males. In a sample of 538 from eight clusters, reported past-year intimate partner violence was halved (adjusted risk ratio 0.45; 0.23, 0.91) [58]. In Uganda, the Safe Homes and Respect for Everyone (SHARE) study added community mobilisation to HIV prevention activities. The intervention involved efforts to change attitudes, social norms, and behaviours related to intimate partner violence. After 3 years, in a sample of 7842 in four intervention and seven control clusters, reported past-year physical (adjusted prevalence rate ratio 0.79; 95% CI 0.67, 0.92) and sexual (0.80; 0.67, 0.7) intimate partner violence were reduced [50]. Also in Uganda, the SASA! intervention recruited community activists to encourage change in norms, attitudes, and behaviours. After about 3 years, in a sample of 2532 in eight clusters, reported past-year physical (odds ratio 0.48; 0.16, 1.39) and sexual (0.76; 0.33, 1.72) intimate partner violence were substantially, but non-significantly, reduced [48, 59]. A couples intervention in Rwanda reduced physical and sexual intimate partner violence in the preceding 12 months [57], and a combination of community mobilisation, group work, and service provision has been trialled in urban Bangladesh [51].

We aim to address known deficiencies in evaluation and partnership. Research on violence against women and girls has been limited, given its ubiquity and health burden, and has favoured description of prevalence and risk factors [5]. Interventional evaluations have tended to focus on high-income countries and on response rather than prevention [23]. Evaluation of the effects of programmes is a priority, particularly through theory-informed testing of interventions to address gender inequality and social norms that legitimise violence [24, 26]. Competent evaluation remains a challenge, non-governmental organisations (NGOs) need to improve their evaluative capacity, and researchers need to partner with implementors [11, 12]. Backed by public engagement, advocacy, and open publication, our vision is of a replicable community-led intervention to address the public health burden of violence against women and girls.

### Ethical considerations

The trial involves data collected on a sensitive subject from women, some of whom will be survivors of violence, in order to evaluate a community intervention that discusses and acts to prevent violence against women. There are three general areas of ethical interest: data collection, intervention, and trial design.

Interviewing women about their possible experience of violence, and men about their views on it, raises issues of consent, interviewer behaviour, privacy and confidentiality, and data sharing. Particular issues we have identified include duty of care after disclosure, breach of confidentiality, veiling of interview content, and perpetration. Duty of care is an issue that we have debated at length, particularly since we do not see a substantial treatment of it in previous research and trials. We feel strongly that an interviewee who discloses experience of violence—physical, sexual, emotional, or gender-based household maltreatment—should be offered optimal support. This goes well beyond presenting her with a list of contact details for local services. Interviewers will be members of a team that is able to provide a full suite of crisis and counselling services, including home visits, medical, surgical, and psychiatric referral, and negotiation with families, the police, and legal representatives.

Although we have not experienced resistance to our community activities in the past 17 years, we assume that the subject of violence against women and girls is not one that communities might readily identify as a problem. Gatekeeper consent is an issue under discussion in cluster trials. The most authoritative review suggests that it is not mandatory [60, 61]. Particular issues are the right of individuals to speak for clusters when the clusters themselves are informal [62], that prominent individuals may not have authority to decide on health issues [63], and that gatekeeper permission implies acceptance of the structural arrangements that underlie gender inequity. Nevertheless, because it is good practice for community interventions, we will seek formal permission from gatekeepers identified by cluster residents. In the same way as for data collectors, we will ensure that community intervention team members are trained and supervised in the ethics of work on violence against women. This extends to confidentiality: group activities are part of the intervention, and facilitators must make it clear to participants that individuals need to be protected.

As with other trials of complex public health interventions, we are testing an intervention that, if successful, will be extended subsequently to control areas. The idea of equipoise will be explained in meetings with cluster gatekeepers. A key issue is benefits to control areas. Our trial differs from other trials, we think, in an important way. Previous trials have tested the effectiveness of community mobilisation and awareness, but have not been grounded in an extensive service network. Our hypothesis is that community mobilisation will offer more benefit than support services alone. We will therefore extend our existing services to cover all clusters, control and intervention. This is similar to the way in which our programme currently supports women and girls who live outside our community action areas. We will make sure that women and girls in these areas have access to all the support agreed by the Council of Europe Istanbul convention [64]. Key compliances achievable at the non-governmental level are condemnation of discrimination against women, prevention through attitudinal and norm change, helping survivors get support through hotlines, shelter, medical, psychological, and legal counselling, protecting women at risk through emergency orders, risk assessment and management, providing support and protection to survivors in judicial proceedings, and collecting epidemiologic and evaluative data.

## Trial status

The protocol is version 9 of 20th March 2019. The protocol was reviewed by the Trial Steering Committee: Seema Sahay (chair), Chris Bonell, Anthony Costello, and Sunita Krishnan. Recruitment began to the baseline survey in early December 2017. Recruitment will be complete for the post-intervention survey in early March 2022. The Standard Protocol Items: Recommendations for Interventional Trials (SPIRIT) checklist is provided as Additional file 4.

## Supplementary information

**Additional file 1.** Questions about effects, and data sources.

**Additional file 2.** Actors in community mobilisation and their roles.

**Additional file 3.** Information sources for context document.

**Additional file 4.** SPIRIT 2013 checklist: recommended items to address in a clinical trial protocol and related documents.

## Data Availability

The final trial dataset will be curated by SNEHA and UCL jointly. Interns and students invited to use data will sign memoranda of understanding and receive anonymised password-protected bespoke datasets. Three kinds of data may be shared. Findings and summary statistics will be shared with SNEHA team members, collaborators, programme participants, media, and the general public. The nature of the output will be decided on a case-by-case basis by project coordinators, the SNEHA Research Group, and the SNEHA directorate. Information on personnel will not be shared except at the request of the individual involved. Anonymised primary datasets will be shared with named investigators, after reduction to relevant variables and password protection.

## Abbreviations

CMO: Context-mechanism-outcome
CTRI: Controlled Trials Registry of India
DAMOCLES: DAta Monitoring Committees: Lessons, Ethics, Statistics
DMEC: Data Monitoring and Ethics Committee
GAD-7: Generalized Anxiety Disorder, 7 item questionnaire
ICC: Intracluster correlation coefficient
ISRCTN: International Standard Registered Clinical/soCial sTudy Number
MIS: Management information system
NFHS: (India) National Family Health Survey
PHQ-9: Patient Health Questionnaire, 9-item
POCSO: (India) Protection of Children from Sexual Offences
PWDVA: (India) Protection of Women from Domestic Violence Act
SNEHA: Society for Nutrition, Education and Health Action
SWEMWBS: Short Warwick-Edinburgh Mental Wellbeing Scale
TARA: Taking Action, Reaching All
TIDieR: Template for Intervention Description and Replication
TMG: Trial Management Group
TSC: Trial Steering Committee
UCL: University College London
WHO: World Health Organization
