# Supplementary material for: Community interventions to prevent violence against women and girls in informal settlements in Mumbai: the SNEHA-TARA pragmatic cluster randomised controlled trial
Source: Trials. 2019 Dec 17;20:743. doi: 10.1186/s13063-019-3817-2 (PMC6918681; doi:10.1186/s13063-019-3817-2)
Supplement: Supplementary file 1 — Additional file 1. Questions about effects, and data sources. [file 13063_2019_3817_MOESM1_ESM.docx]

# Table S1. Questions about effects, and data sources

| **Questions based on theory of change** | **Source of data** | | | | | |
| --- | --- | --- | --- | --- | --- | --- |
|  |  | | | | | |
| **Changes:** **survivors of VAWG identified and supported** | **3 year experience survey** | **3 year community attitudes survey** | **Intervention MIS** | **Interviews, observation** | **Case cohort** | **Periodic assessment and 1.5 year survey** |
| Were survivors identified? |  |  |  |  |  |  |
| Were survivors counseled? |  |  |  |  |  |  |
| Were survivors referred? |  |  |  |  |  |  |
| Did survivors access medical, police, and legal services? |  |  |  |  |  |  |
| Did survivors come to understand the nature of VAWG and take action to prevent or respond to it? |  |  |  |  |  |  |
| Did perpetrators come to understand abuse and others’ concerns? |  |  |  |  |  |  |
| Did abuse cease to be private such that others came to know about it? |  |  |  |  |  |  |
| Did survivors’ families and friends seek help for VAWG? |  |  |  |  |  |  |
| Did survivors feel less alone and more empowered? |  |  |  |  |  |  |
| Did survivors’ mental health improve? |  |  |  |  |  |  |
| Did survivors change their home situations? |  |  |  |  |  |  |
|  |  |  |  |  |  |  |
| **Changes: programme participants change** | **3 year experience survey** | **3 year community attitudes survey** | **Intervention MIS** | **Interviews, observation** | **Case cohort** | **Periodic assessment and 1.5 year survey** |
| Did people join groups or form more groups and networks? |  |  |  |  |  |  |
| Did groups and networks have collective agency? |  |  |  |  |  |  |
| Did people discuss gender roles and VAWG? |  |  |  |  |  |  |
| Did people develop confidence to challenge norms? |  |  |  |  |  |  |
| Did people become leaders or change agents and bear witness to VAWG? |  |  |  |  |  |  |
| Did people trust SNEHA, police, legal, and medical services? |  |  |  |  |  |  |
| Did people demand implementation of the law? |  |  |  |  |  |  |
|  |  |  |  |  |  |  |
| **Changes: communities change** | **3 year experience survey** | **3 year community attitudes survey** | **Intervention MIS** | **Interviews, observation** | **Case cohort** | **Periodic assessment and 1.5 year survey** |
| Did events lead to enquiries and awareness of program activities? |  |  |  |  |  |  |
| Did communities identify and report VAWG, and did referrals for early intervention increase? |  |  |  |  |  |  |
| Did communities support women and impose sanctions against VAWG? |  |  |  |  |  |  |
| **Outcomes** | **3 year experience survey** | **3 year community attitudes survey** | **Intervention MIS** | **Interviews, observation** | **Case cohort** | **Periodic assessment and 1.5 year survey** |
|  |  |  |  |  |  |  |
| Did survivors disclose VAWG? |  |  |  |  |  |  |
| Were communities less tolerant of VAWG? |  |  |  |  |  |  |
| Did attitudes to rape and sexual violence change? |  |  |  |  |  |  |
| Did attitudes to gender roles change? |  |  |  |  |  |  |
| Did bystander attitudes change? |  |  |  |  |  |  |
| Did bystander intervention increase? |  |  |  |  |  |  |
| Did prevalence of non-partner sexual violence decrease? |  |  |  |  |  |  |
| Did domestic physical and sexual violence decrease? |  |  |  |  |  |  |
| Did domestic emotional, economic, control and neglect decrease? |  |  |  |  |  |  |
|  |  |  |  |  |  |  |
| **Adverse effects** | **3 year experience survey** | **3 year community attitudes survey** | **Intervention MIS** | **Interviews, observation** | **Case cohort** | **Periodic assessment and 1.5 year survey** |
| Did violence increase? |  |  |  |  |  |  |
| Did vigilantism and precipitate action occur? |  |  |  |  |  |  |
| Were limits set to women’s mobility? |  |  |  |  |  |  |
| Were survivors of VAWG or the wrong people punished? |  |  |  |  |  |  |
| Did perpetrators switch type of VAWG? |  |  |  |  |  |  |
| Were there threats or violence against sanginis, activists or families? |  |  |  |  |  |  |
| Did families or communities militate against sanginis or SNEHA? |  |  |  |  |  |  |
| Did emerging leaders have their own agendas that conflicted with the program agenda? |  |  |  |  |  |  |
| Did emerging voice leas to favoritism and corruption? |  |  |  |  |  |  |

VAWG: violence against women and girls. MIS: management information system.
