# Supplementary material for: Community interventions to prevent violence against women and girls in informal settlements in Mumbai: the SNEHA-TARA pragmatic cluster randomised controlled trial
Source: Trials. 2019 Dec 17;20:743. doi: 10.1186/s13063-019-3817-2 (PMC6918681; doi:10.1186/s13063-019-3817-2)
Supplement: Supplementary file 2 — Additional file 2. Actors in community mobilisation and their roles. [file 13063_2019_3817_MOESM2_ESM.docx]

# Table S2. Actors in community mobilization and their roles

|  | **Indicator** | **Source of data** |
| --- | --- | --- |
| **Community mobilization and group formation** |  |  |
| **Community organisers and officers** | Characteristics of staff | Recruitment information |
| Convene groups | Groups formed  Mobilisation  Meeting number  Meeting regularity  Attendance | Intervention MIS  Periodic assessment  Intervention MIS  Intervention MIS  Intervention MIS |
| Deliver modules according to protocol | Modules delivered | Intervention MIS |
|  | Modules to time | Intervention MIS |
| Facilitate group functionality | Group actions | Intervention MIS |
|  | Quality of facilitation | Intervention MIS  Interviews, observation |
|  | Quality of supervision | Intervention MIS  Interviews, observation |
| **Group members** | Characteristics of group members | Intervention MIS |
| Attend groups | Attendance total and regularity | Intervention MIS |
| Participate in modules | Quality of participation | Intervention MIS  Interviews, observation |
| Develop group functionality | Group dynamics | Intervention MIS  Interviews, observation |
|  | Group action | Intervention MIS |
| **Sanginis** |  |  |
| Show interest and leadership qualities | Emergent leadership | Intervention MIS  Interviews, observation |
|  | Sangini selection | Intervention MIS  Interviews, observation |
|  |  |  |
|  |  |  |
| **Crisis intervention, counseling, and support for survivors** |  |  |
| **Community organisers and officers** | **Indicator** | **Source of data** |
| Provide support to survivors of VAWG, coordinate family interventions, arrange referral to SNEHA, police or health services, and coordinate and undertake community follow-up | Survivors identified | Intervention MIS |
|  | Family interventions | Intervention MIS  Interviews, observation |
|  | Referrals to SNEHA, police, health services | Intervention MIS |
|  | Survivor follow-up | Intervention MIS |
| Act as main contact between community and counseling teams, and communicate with stakeholder networks | Communication between sangini and counselor | Interviews, observation |
|  | Communication with stakeholder networks | Intervention MIS  Interviews, observation |
| Support sanginis in their field activities | Support for sangini work with survivors | Interviews, observation |
| **Group members** |  |  |
| Identify incidences of VAWG and inform sanginis and community officers. | Survivors identified | Intervention MIS |
|  | Sanginis informed by group members | Intervention MIS |
|  | Community organisers and officers informed by group members | Intervention MIS |
| Intervene to ameliorate conflict, arrange referral to SNEHA, support survivors of VAWG in accessing family interventions and police or health services, locate perpetrators of VAWG and negotiate with them and families. | Local interventions | Intervention MIS |
|  | SNEHA referrals | Intervention MIS |
|  | Family interventions | Intervention MIS |
|  | Police referrals | Intervention MIS |
|  | Health service referrals | Intervention MIS |
|  | Perpetrators and families communicated with | Intervention MIS  Interviews, observation |
| **Sanginis** |  |  |
| Identify incidences of VAWG, assess safety, provide initial counseling and information on rights and law to survivors, negotiate action, record incidents with Little Sister, intervene to ameliorate conflict, arrange referral to SNEHA, organize temporary shelter and childcare. | Survivors identified | Intervention MIS |
|  | Safety assessments | Intervention MIS |
|  | Initial counseling | Intervention MIS |
|  | Planned action | Intervention MIS |
|  | Little Sister registration | Intervention MIS |
|  | Crisis intervention | Intervention MIS |
|  | SNEHA referrals | Intervention MIS |
|  | Shelter arrangement | Intervention MIS |
|  | Childcare arrangement | Intervention MIS |
| Support survivors of VAWG in accessing family interventions and police or health services, locate perpetrators of VAWG and negotiate with them and families, and conduct community follow-up. | Family interventions | Intervention MIS |
|  | Police referrals | Intervention MIS |
|  | Health service referrals | Intervention MIS |
|  | Perpetrators and families communicated with | Intervention MIS  Interviews, observation |
|  | Survivor follow-up | Intervention MIS |
| **Counselors** |  |  |
| Provide crisis counseling and intervention services, counsel survivors of VAWG and their families | Crisis interventions | Intervention MIS |
|  | Counseling delivered | Intervention MIS |
| Make home visits for crisis intervention, family discussions, and follow-up | Home visits | Intervention MIS |
| Organise referral to police, health and legal services and negotiate with them. | Police referrals | Intervention MIS |
|  | Health service referrals | Intervention MIS |
|  | Legal referrals | Intervention MIS |
| Assess mental health and refer for therapy | Mental health assessments | Intervention MIS |
|  | Psychologist referrals | Intervention MIS |
| **Community action and system liaison** | **Indicator** | **Source of data** |
| **Community organisers and officers** |  |  |
| Organise and participate in community campaigns and support visible collective action | Campaign number | Intervention MIS |
|  | Campaign and event attendance | Intervention MIS |
|  | Number of attenders who stayed | Intervention MIS |
|  | Number of requests for information | Intervention MIS |
|  | Number of requests for help | Intervention MIS |
| Liaise with police and health providers and negotiate with community bodies | Number of outreach discussions | Intervention MIS |
| **Group members** | Group emotional intelligence etc | Periodic assessment |
| Participate in community campaigns and contribute to visible collective action | Number of collective interventions | Intervention MIS |
|  | Types of collective intervention | Intervention MIS |
|  | Number of group members networking | Intervention MIS |
|  | Type of networking | Intervention MIS |
|  | Number of group members discussing gender norms | Intervention MIS |
|  | Type of people discussing gender norms | Intervention MIS |
|  | Number of individual interventions | Intervention MIS |
| Liaise with police and health providers and negotiate with community bodies | Police referrals | Intervention MIS |
|  | Health service referrals | Intervention MIS |
|  | Community body discussions | Intervention MIS |
| Negotiate with municipal representatives for infrastructure and entitlements | Corporator discussions | Intervention MIS |
| **Sanginis** |  |  |
| Support and participate in community campaigns and contribute to visible collective action | Sangini presence at events  Collective action organisation | Intervention MIS  Intervention MIS |
| Liaise with police and health providers and negotiate with community bodies | Police referrals | Intervention MIS |
|  | Health service referrals | Intervention MIS |
|  | Community body discussions | Intervention MIS |

MIS: management information system.
