# Supplementary material for: Community interventions to prevent violence against women and girls in informal settlements in Mumbai: the SNEHA-TARA pragmatic cluster randomised controlled trial
Source: Trials. 2019 Dec 17;20:743. doi: 10.1186/s13063-019-3817-2 (PMC6918681; doi:10.1186/s13063-019-3817-2)
Supplement: Supplementary file 3 — Additional file 3. Information sources for context document. [file 13063_2019_3817_MOESM3_ESM.docx]

# Webtable 3. Information sources for context document

| **Context document** | **Source of data** | | | | |
| --- | --- | --- | --- | --- | --- |
|  | **Microplanning** | **Cluster mapping and vulnerability assessment** | **Baseline experience survey** | **Baseline community attitudes survey** | **Interviews, observation** |
| History of phase and cluster areas |  |  |  |  |  |
| Commonalities and differences between clusters |  |  |  |  |  |
| Urban space in clusters |  |  |  |  |  |
| Candidate constructs for use in hypothesis development and testing |  |  |  |  |  |
